# Antagonistic Strain *Bacillus amyloliquefaciens* XZ34-1 for Controlling *Bipolaris sorokiniana* and Promoting Growth in Wheat

**DOI:** 10.3390/pathogens10111526

**Published:** 2021-11-22

**Authors:** Yanjie Yi, Youtian Shan, Shifei Liu, Yanhui Yang, Yang Liu, Yanan Yin, Zhipeng Hou, Pengyu Luan, Ruifang Li

**Affiliations:** 1 School of Biological Engineering, Henan University of Technology, Zhengzhou 450001, China; shanyoutian@stu.haut.edu.cn (Y.S.); 201720040312@stu.haut.edu.cn (S.L.); yyhui2004@haut.edu.cn (Y.Y.); liuyang01@stu.haut.edu.cn (Y.L.); christa@stu.haut.edu.cn (Y.Y.); 2021920170@stu.haut.edu.cn (Z.H.); 2021920159@stu.haut.edu.cn (P.L.); 2 The Key Laboratory of Functional Molecules for Biomedical Research, Zhengzhou 450001, China

**Keywords:** *Bacillus amyloliquefaciens*, *Bipolaris sorokiniana*, common root rot, biological control, growth promoting

## Abstract

Common root rot, caused by *Bipolaris sorokiniana*, is one of the most prevalent diseases of wheat and has led to major declines in wheat yield and quality worldwide. Here, strain XZ34-1 was isolated from soil and identified as *Bacillus amyloliquefaciens* based on the morphological, physiological, biochemical characteristics and 16S rDNA sequence. Culture filtrate (CF) of strain XZ34-1 showed a high inhibition rate against *B.* *sorokiniana* and had a broad antifungal spectrum. It also remarkably inhibited the mycelial growth and spore germination of *B. sorokiniana*. In pot control experiments, the incidence and disease index of common root rot in wheat seedlings were decreased after treatment with CF, and the biological control efficacy was significant, up to 78.24%. Further studies showed XZ34-1 could produce antifungal bioactive substances and had the potential of promoting plant growth. Lipopeptide genes detection with PCR indicated that strain XZ34-1 may produce lipopeptides. Furthermore, activities of defense-related enzymes were enhanced in wheat seedlings after inoculation with *B.* *sorokiniana* and treatment with CF, which showed induced resistance could be produced in wheat to resist pathogens. These results reveal that strain XZ34-1 is a promising candidate for application as a biological control agent against *B.* *sorokiniana*.

## 1. Introduction 

Common root rot is one of the most prevalent diseases of wheat and has become a severe threat to global wheat production [1]. It is characterized by the development of necrotic lesions on the roots and sub-crown. The lesions are dark brown to black in color. The development of symptoms on the roots is usually followed by symptoms on the crowns of wheat [2,3,4]. Common root rot has been reported in many wheat-growing regions, such as China, North America, the Middle East, Australia, etc. [5,6,7]. As the pathogenic fungi of wheat common root rot, *Bipolaris sorokiniana* results from soil-borne or seed-borne inoculum and can attack all wheat parts, including seeds, roots, shoots, and leaves, and results in yield losses in wheat [2]. When severely infected, it can also cause the aboveground parts of the wheat to die. In addition, it seriously affects the quality and nutrition of wheat flour [8].

Cultural management, resistant varieties and fungicides are the most practices to control common root rot of wheat. Cultural management is important for controlling plant diseases. The rotation of wheat with cruciferous crops and soil solarization were found to reduce the incidence of common root rot [9,10]. Zero tillage with rotation and residue retention reduced the incidence of root rot of wheat in Mexico, but they are difficult to implement in China due to the large population and limited arable land [11]. Fewer completely resistant wheat varieties have been reported in traditional breeding [2]. Although chemical fungicides have become an important strategy for common root rot control, they create imbalances in the microbial community, which may have adverse effects on beneficial microorganisms and lead to the development of resistant pathogens [12,13].

Exploiting biocontrol agents has acquired intense attention nowadays. It is important to find antagonistic strains that can complement cultural and chemical practices in the field [2]. In recent years, biological control has been used in the control of common root rot of wheat. Some antagonistic strains (e.g., *Pseudomonas mediterranea,* *Pseudomomas fluorescens, Nocardiopsis dassonvillei, Lysobacter enzymogenes and Chaetomium globosum,* et al.) were found to inhibit *B. sorokiniana* [14,15,16,17]. Among the antagonistic bacteria, *Bacillus* spp. are widely present in soil and are considered to have the highest biocontrol potential, being of greater interest than other species, and their long-term viability is more conducive to the development of commercial products. Many studies have reported that using *Bacillus* strains as biological control agents are an effective and safe control strategy [16,17,18]. *Bacillus* spp. can form endospores to resist hostile conditions, thus giving *Bacillus*-based products an advantage over other biocontrol microorganisms [18]. Up to now, more than 100 commercial products of *Bacillus* preparations have been registered in China [19]. Among the *Bacillus* species, *B. amyloliquefaciens* are known for their ability to promote plant growth and fight a wide range of plant-associated diseases, and they are considered promising biocontrol agents [18,19]. Many studies have reported that *B. amyloliquefaciens* could control diseases caused by a variety of pathogens [20,21,22,23]. For example, *B. amyloliquefaciens* S76-3 produces three classes of cyclic lipopeptides, including Iturin, Plipastatin and Surfactin, which exhibit strong antifungal activity against the toxigenic *Fusarium graminearum* [22]. Furthermore, *B. amyloliquefaciens* has shown the ability to promote plant growth [24,25]. The potential contribution to plant growth promotion by *B. amyloliquefaciens* UCMB5113 is its ability to produce IAA, acetoin and siderophore [26].

To obtain more effective strains and further investigate their actions for exploiting microbial agents to control wheat root rot, in the present work, strain XZ34-1 antagonistic to *B. sorokiniana* was isolated and identified, and its biological control effect, antifungal mechanism and plant growth-promoting characteristics were evaluated.

## 2. Results

### 2.1. Screening of Antagonistic Bacteria

A total of 81 bacteria were isolated from the soil of the Tibetan plateau. All of them were tested for inhibition on pathogenic fungi *B.* *sorokiniana* by the plate confrontation method, and the five most effective antagonistic isolates were selected. The Oxford cup method was used to test the antagonistic effect of five isolates against the pathogenic fungi, and the results showed that XZ34-1 had the strongest antifungal effect and was selected for further studies. The inhibition rates of XZ34-1 culture filtrate (CF) on *Bipolaris* *sorokiniana*, *Fusarium graminearum*, *Rhizoctonia zeae*, *Aspergillus niger* and *Aspergillus flavus* were 53.52, 43.06, 46.70, 35.77 and 34.35%, respectively (Table 1).

### 2.2. Identification of Antagonistic Strain XZ34-1

**Figure 1:**
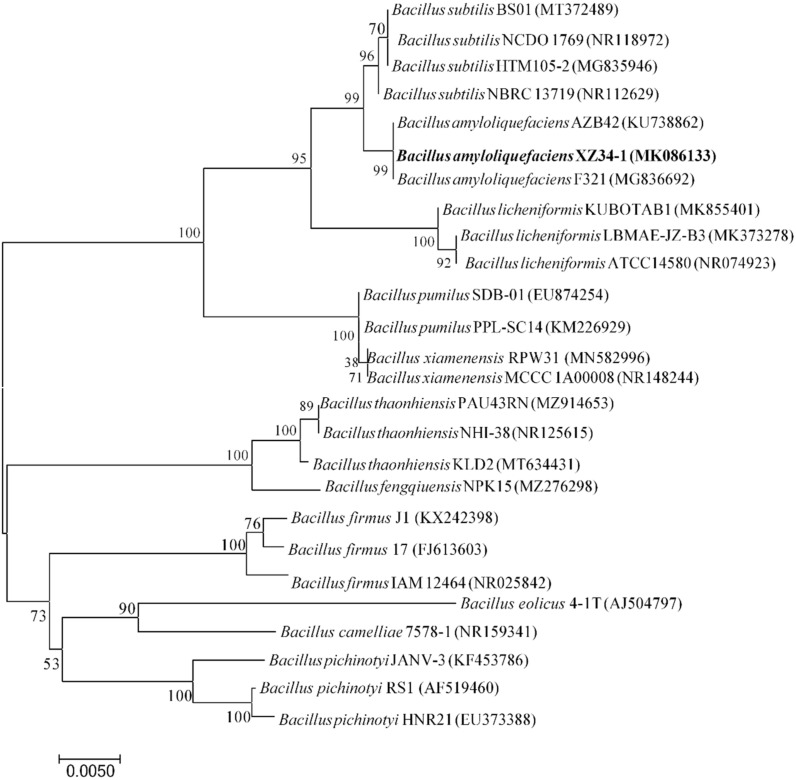
Phylogenetic tree constructed based on 16S rDNA sequences. The analysis was performed using the Neighbor-Joining method of MEGA 7.0 (1000 bootstrap values).

Strain XZ34-1 is Gram-positive and forms endospores. Pure colonies of XZ34-1 after 48 h incubation showed creamy white in appearance without pigmentation, rough surface, rounded overall, smooth margins and opaque. The bacteria produce acids, not gases, from glucose decomposition. The V-P test, the tests of inorganic and organic phosphorus dissolving, siderophore production, nitrogen fixation, protease and pectinase production are all positive (Table 2). It indicated that the isolate XZ34-1 belonged to *Bacillus* spp. Based on the 16S rDNA sequence alignments, a phylogenetic tree was constructed (Figure 1). The strain XZ34-1 showed the highest homology (98.25%) with *B. amyloliquefaciens* F321 (MG836692), and the strains were clustered on the same evolutionary branch as *B. amyloliquefaciens*. According to morphological, physical and chemical characteristics and molecular analysis, the strains XZ34-1 were identified as *B. amyloliquefaciens*.

### 2.3. Inhibitory Effect of the Antagonistic Strain XZ34-1 CF on B. sorokiniana

**Figure 2:**
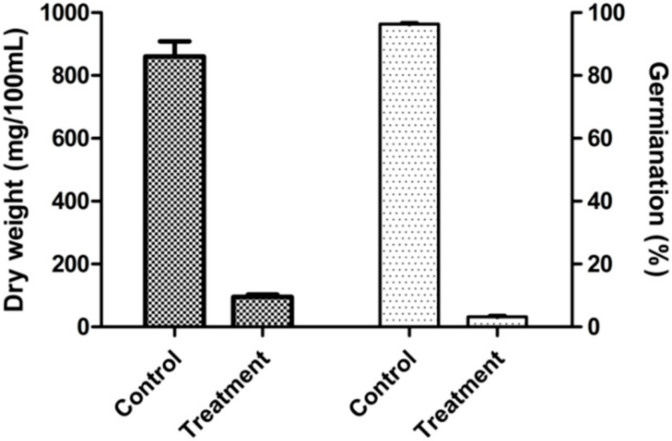
Inhibition effect of XZ34-1 CF on *B.* *sorokiniana*. Error bar represents the standard error of the means of replicates (*n* = 10).

Inhibitory rates of the antagonistic strain XZ34-1 CF on *B.* *sorokiniana* were detected by evaluating mycelial dry weight and spore germination. The results showed the CF of strain XZ34-1 could remarkably inhibit the mycelial growth and spore germination of *B.* *sorokiniana* and indicated that active metabolites might be present in the CF of strain XZ34-1 (Figure 2).

### 2.4. Biocontrol Efficacy of XZ34-1 CF on Common Root Rot Caused by B. sorokiniana

**Figure 3:**
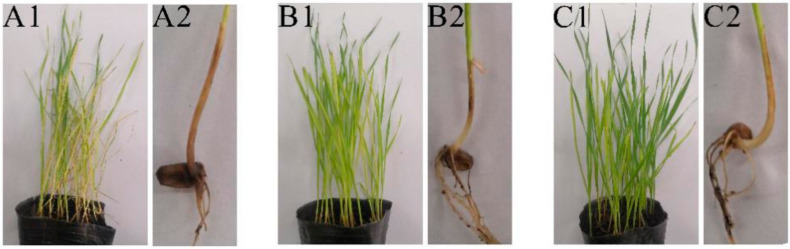
Biocontrol efficiency of XZ34-1 CF on common root rot caused by *B.* *sorokiniana*. The pathogen *B. sorokiniana* was pre-inoculated on all of the wheat seedlings tested. (**A1**,**A2**) were sterile water treatment (control); (**B1**,**B2**) were 50% carbendazim treatment; (**C1**,**C2**) were XZ34-1 CF treatment.

The biocontrol efficacy of strain XZ34-1 against *B. sorokiniana* was evaluated using wheat seedlings in pots. Figure 3A1,A2 showed that wheat seedlings wilted, and the roots developed necrotic lesions (dark brown) after being infected with *B. sorokiniana*. Wheat seedlings returned to normal after treatment with 50% carbendazim and XZ34-1 CF (Figure 3B1,B2,C1,C2). Compared with the positive control (50% carbendazim) and the negative control (sterile water), CF of XZ34-1 exhibited significant biological control efficacy 78.24% on the incidence of common root rot in wheat seedlings; however, this was lower than carbendazim (Table 3). These results indicated XZ34-1 CF has great application value.

### 2.5. Potential of Strain XZ34-1 in Fungal Antagonism and Growth Promotion

The potential of strain XZ34-1 for antagonizing fungi and promoting plant growth was evaluated by characterizing several indicators on a plate. The results showed that XZ34-1 had the potential to decompose organic phosphorus and inorganic phosphorus and to fix nitrogen. However, the nitrogen-fixation ability of the strain XZ34-1 should be confirmed by PCR amplification of nifH gene encoding for nitrogenase activity. In addition, it could secrete protease, pectinase and siderophore. However, it could not produce cellulase and chitinase. For IAA (Indole acetic acid) detection, the pink color did not appear, indicated that IAA was not produced by strain XZ34-1.

### 2.6. Detection of Lipopeptide Biosynthesis Genes of Strain 34-1

Strain XZ34-1 contains genes involved in lipopeptide biosynthesis, which can be detected by PCR method with primer pairs designed to detect genes involved in Bacyllomicin (BMYBa, BMYBb), Bacilysin (BACa, BACb), Bacillaene (BAE), Iturin (ITU), Fengycin (fenD) and Surfactin (srfAA, srfP) biosynthesis. As shown in Table 4, amplified fragments of the expected size of Bacyllomicin, Bacilysin, Bacillaene and Iturin were obtained. Therefore, lipopeptides may be produced by antagonistic strain XZ34-1 and are the active substances partially responsible for the antifungal activity.

### 2.7. Effect of XZ34-1 CF on Cell Membrane Integrity in B. sorokiniana

**Figure 4:**
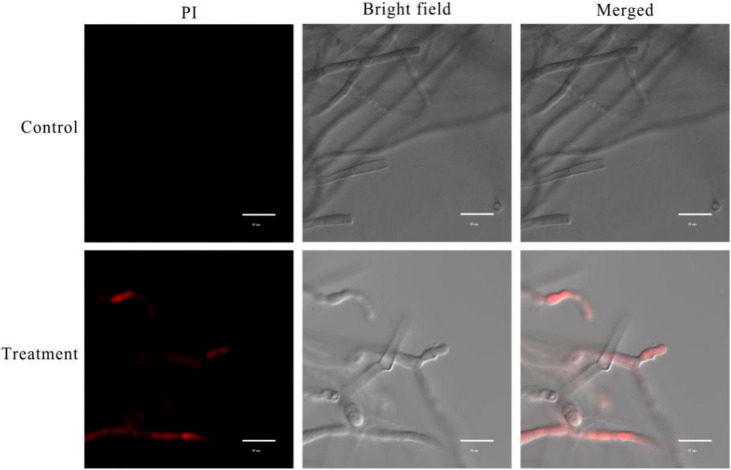
Effects of XZ34-1 CF on the morphology and cell membrane integrity of *B. sorokiniana*. Scale bar, 15 μm.

After staining by propidium iodide (PI), the mycelia of *B. sorokiniana* with and without CF treatment were observed using a laser scanning confocal microscope (LSCM). The surface of mycelia treated by CF became rough, and the tip was swollen. In addition, PI staining showed that obvious red fluorescence was present in the mycelia treated by CF, while it was absent in the control mycelia (Figure 4). These results indicated that the membrane integrity of mycelia was destroyed by CF of antagonistic strain XZ34-1.

### 2.8. Effects of XZ34-1 CF on Antioxidant Activity of Wheat Seedlings

**Figure 5:**
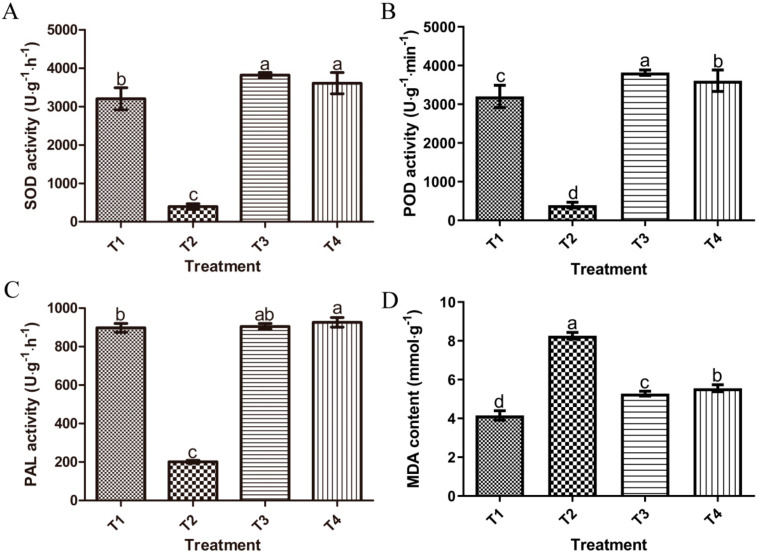
Superoxide dismutase (SOD), peroxidase (POD) and phenylalanine ammonia lyase (PAL) activities and malondialdehyde (MDA) content in wheat seedlings. T1 (control, normal-grown wheat seedlings), T2 (inoculation with *B. sorokiniana*), T3 (carbendazim treatment after inoculation with *B. sorokiniana*) and T4 (XZ34-1 CF treatment after inoculation with *B. sorokiniana*). (**A**) = SOD activity, (**B**) = POD activity, (**C**) = PAL activity, (**D**) = MDA content. The data presented are the mean value of at least five repeats. Letters on bars indicate DMRT at *p* < 0.05.

Activities of superoxide dismutase (SOD), peroxidase (POD), phenylalanine ammonia lyase (PAL) and malondialdehyde (MDA) concentrations in the root tissue extracts of wheat were detected after inoculating *B. sorokiniana* and treatments with XZ34-1 CF and 50% carbendazim. Figure 5A–C showed that the antioxidant enzyme activities in both the XZ34-1 CF-treated group (T4) and the 50% carbendazim-treated group (T3) were higher and even surpassed those of normal-grown wheat seedlings (T1) compared to the inoculation with *B. sorokiniana* only (T2). The SOD, POD and PAL activities in the T4 group were significantly increased by 2.0, 9.1 and 4.5-fold, respectively, compared to the T2 group. MDA is one of the most important products of membrane lipid peroxidation. Therefore, the degree of membrane lipid peroxidation can be reflected by MDA content and can indirectly reflect the damage degree of the membrane system. Figure 5D revealed that the content of MDA in the T4 group was significantly lower than that in the T2 group, only 67.2% of its concentration.

## 3. Discussion

Common root rot reduces wheat yield and causes huge economic losses. *B.* *sorokiniana* is soil-borne or seed-borne, and once conditions are favorable, it develops rapidly and infects plants. Eber Villa-Rodríguez et al. reported that *Bacillus subtilis* TE3 could inhibit *B. sorokiniana* mycelia dry weight through culture filtrate [34]. Filtrate of liquid culture of *Chaetomium* spp. strain 22-10 also inhibited the mycelial growth of *B.* *sorokiniana*, indicating that strain 22-10 produced secondary metabolites against *B.* *sorokiniana* [16]. In the current study, 81 strains of bacteria were isolated from soil, and five of them exhibited strong antagonistic potential against *B.* *sorokiniana*. Strain XZ34-1 was identified as *B. amyloliquefaciens* and showed a high inhibition rate against pathogenic *B.* *sorokiniana*. We investigated the antifungal activities of CF of XZ34-1 against *B.* *sorokiniana* and found that the CF can inhibit mycelial dry weight and conidial germination. The pot control experiments showed that CF of *B.* *amyloliquefaciens* XZ34-1 had a significant influence on common root rot of wheat caused by *B. sorokiniana*, indicating the higher potential of XZ34-1 as a biological agent to control *B. sorokiniana* in wheat.

The antifungal mechanisms by *Bacillus* to control plant diseases and induce plant growth differ due to differences in bacterial species. Therefore, understanding the biocontrol mechanism of *B. amyloliquefaciens* XZ34-1 will help us to evaluate and improve the biocontrol effect of *B.* *sorokiniana* in wheat crops. The current work indicates that *B. amyloliquefaciens* XZ34-1 exhibits plant growth-promoting traits. Strain XZ34-1 was able to grow in a nitrogen-free medium and indicated its potential to fix atmospheric N_2_. Zakry, F. A. et al. reported that *Bacillus* *sphaericus* strain UPMB-10 has the nifH gene and produces nitrogenase (EC 1.18.6.1), which can fix atmospheric N_2_ and provide it to plants to enhance plant growth and yield [35]. Strain XZ34-1 was also able to solubilize organic and inorganic phosphorus, which is an important feature of enhancing soil fertility and plant growth. The study by Muhammad Tahir et al. showed that the combined application of bio-organic phosphate and phosphorus-solubilizing bacteria MWT 14 could harvest better wheat yield with low fertilizer inputs under an arid climate [36]. The ability of siderophore production in strain XZ34-1 was confirmed by a CAS-blue agar assay. Similarly, Saoussen Ben Khedher et al. revealed that *B. subtilis* V26 was able to produce siderophore [32]. Siderophores have an exceptionally high affinity for Fe^3+^ and are able to bind the Fe-siderophore complexes, thus promoting Fe uptake by microorganisms, while the complexes can also be used by plants to increase iron content inside plant tissues and improve plant growth [37]. *Nocardiopsis dassonvillei* as a biocontrol agent can control wheat common root rot and enhance the growth of wheat attributed to the ability to produce siderophores, hydrogen cyanide and IAA, which are commonly known features for the plant-growth promotion and biocontrol activities of a given strain [15].

The control of plant pathogens by *Bacillus* is closely related to its ability to secrete hydrolytic enzymes. Antimicrobial activity through the production of fungal cell wall degrading enzymes is probably an important mechanism used by biocontrol bacteria to inhibit pathogens. Lopes et al. reported that six *S. cerevisiae* isolates could inhibit pathogen germination, produce killer activity and hydrolytic enzymes when in contact with the fungus wall [38]. The current study demonstrated the ability of strain XZ34-1 to produce protease and pectinase; however, no cellulase or chitinase production was detected. It indicated the protease and pectinase might limit the pathogen invasion into host wheat tissues. Several reports showed that such enzymes seem to be stable enough for semi-field or field applications, as their range of activity appears adequate to inhibit fungal phytopathogens growing in acidic and mesophilic environments [39,40].

Another important mechanism by *Bacillus* for suppressing plant pathogens is the production of antifungal metabolites and antibiotics [41,42,43,44,45]. The biocontrol fungus *Chaetomium globosum* is effective in inhibiting *B. sorokiniana* associated with wheat common root rot owing to the production of secondary metabolites by *C. globosum* [16]. Six genes involved in lipopeptide biosynthesis were detected by PCR in strain XZ34-1. The results indicated XZ34-1 could produce Bacyllomicin, Bacilysin, Bacillaene and Iturin. These antifungal metabolites have well-recognized potential uses in biocontrol [32]. *Bacillus velezensis* AK-0 was shown to co-produce the lipopeptides iturin and bacilysin, which have potent antifungal effects against bitter rot in apples [46]. Bacillomycin L and Fengycin A compounds were isolated from strain G341, which controlled the development of rice blast, sheath blight, tomato gray mold and red pepper anthracnose [47]. Lipopeptides from *Bacillus megaterium* strain WL-3 containing three subfamilies, surfactant, Iturin A and Fengycin A, which control late blight and promote potato plant growth [43]. In our opinion, it is necessary to assay and evaluate the lipopeptide of XZ34-1 in the future to further explore its mechanism of action and broaden its application.

Maintaining the stability of defense systems in the host plant is a promising mechanism for effective biological control of fungal pathogens [48]. This study showed that CF-treated wheat seedlings were observed increasing on SOD, POD and PAL activities and MDA content compared to normally grown wheat. It indicated the degree of damage to the membrane system was significantly lower than that of wheat inoculated with *B. sorokiniana* only. Several reports have shown that the strains can directly trigger plant defense mechanisms by themselves and induce the plant’s self-defense systems in response to an external attack by fungal pathogens [16,35,36]. In our results, the SOD, POD and CAT activities were significantly enhanced, and the MDA content was significantly reduced in the treated diseased seedlings. As shown in Figure 3, the treated wheat has returned to normal growth. Moreover, CF-treated and carbendazim-treated wheat seedlings were observed increasing obviously on SOD, POD and PAL activities compared to normal-grown seedlings. Since CF treatment and carbendazim treatment caused oxidative stress in the seedlings and in order to scavenge harmful reactive oxygen species, the enzymatic antioxidative defense system of wheat becomes activated. This resulted in enhanced activities of antioxidative enzymes. The internal defense system of plants helps to protect them from oxidative stress caused by stressful conditions [48,49,50,51]. Based on these data, we speculate the CF of XZ34-1 controls the common root rot caused by *B.* *sorokiniana* by producing some antibiotic substances to prevent pathogen infection, altering plant physiological and metabolic responses, stabilizing plant cell membranes and rejuvenating plants after pathogen infection. In the future, field applications should be conducted to investigate the biocontrol effects of XZ34-1 CF against common root rot of wheat to support the development of commercial formulation.

## 4. Materials and Methods

### 4.1. Materials

Soil samples used for isolating the antagonistic bacteria were provided by Dr. Yu Shi of the Institute of Soil Science, Chinese Academy of Sciences. Pathogenic fungi *B.* *sorokiniana*, *Fusarium graminearum*, *Rhizoctonia zeae*, *Aspergillus niger* and *Aspergillus flavus*) were kept at −80 ± 2 °C in 15% glycerol stocks in the School of Biological Engineering, Henan University of Technology. The chemical fungicide carbendazim was purchased from the local market. Wheat varieties used in pot control were Qiule 2122 (purchased from Henan Qiule Seeds Technology, Co., Ltd., Zhengzhou, China). The medium was LB medium (5 g yeast extract, 10 g peptone, 10 g NaCl, 15–20 g agar, 1000 mL distilled water, pH 7) and potato dextrose agar (PDA) medium (20 g glucose, 200 g potato, agar 15–20 g agar, 1000 mL distilled water, pH 7).

### 4.2. Isolation and Screening of Antagonistic Bacteria

Soil samples were collected from different locations on the Tibetan Plateau. Soil samples were transported to the laboratory until strains of bacteria were isolated using the serial dilution method [19,52,53]. The strains with better antagonistic effects were selected and the Oxford cup method was used to determine their inhibition activities [54]. Briefly, the antagonistic bacteria were adjusted to a concentration of 10^8^ cfu mL^−1^ and inoculated into conical flasks (250 mL) containing 100 mL LB liquid medium at 1% inoculum, which was cultured at 37 °C with shaking at 180 r min^−1^ for 72 h. The fermentation broth was centrifuged at 8000 r min^-1^ for 15 min and filtered through a 0.22 μm pore size membrane filter to obtain a culture filtrate (CF). Disks containing pathogenic fungi were placed in the center of the PDA plate, then an Oxford cup was placed 30 mm from the center, and 200 μL of CF was transferred into the cup. The PDA plate without CF was set as a control group, and the same pathogenic fungi were cultured for the same period of time. Nine replicate plates were maintained for each CF. The inhibitory rate was calculated following the formula: inhibitory rate (%) = (C − T)/C × 100%, where C is the diameter of the control fungus and T is the diameter of the treatment fungus. The strain showing the strongest antifungal effect was coded as XZ34-1 and preserved in 40% glycerol on the slope of the test tube at –80 °C for further processing [19].

### 4.3. Identification of Strain XZ34-1

#### 4.3.1. Morphological, Physiological and Biochemical Tests

Antagonistic strain XZ34-1 was cultured on LB medium plate and placed at 28 °C for 24 h to observe colony characteristics, such as color, morphology and growth properties. Physiological and biochemical tests were performed using universal methods [55], including gram staining, nitrate reduction, citrate utilization, Voges-Proskauer (V-P) reaction, methyl red and glucose decomposition test.

#### 4.3.2. Molecular Identification of Antagonistic Strain XZ34-1

Genomic DNA of XZ34-1 was extracted using the phenol-chloroform method [35]. The 16S rDNA gene was amplified using the bacterial universal primer pair 16SF (5’-AGA GTT TGA TCA TGG CTC AG-3’) and 16SR (5’-ACG GTT ACC TTG TTA CGA CTT-3’) primers [34,56]. PCR was performed using a Taq DNA polymerase kit (Sangon Biotech Co., Ltd., Shanghai, China) in 25 μL reactions. The PCR conditions were as follows: 94 °C for 5 min, followed by 32 cycles of 30 s at 94 °C, 30 s at 55 °C and 90 s at 72 °C, then a final extension step of 10 min at 72 °C. Then the PCR product was sent to Sangon Biotech Co., Ltd. (Shanghai, China) for sequencing. The 16S rDNA sequence of XZ34-1 was submitted to GenBank (NCBI, USA; http://www.Ncbi.nlm.nih.gov) under the accession number MK086133. The DNA sequences were blasted in the GenBank, and the phylogenetic tree was constructed with MEGA 7.0 software using the Maximum Likelihood method with 1000 bootstrap replications [57].

### 4.4. Inhibitory Effect of the Antagonistic Strain XZ34-1 CF on B. sorokiniana

Spore suspension (1 × 10^6^ cfu/mL) of *B. sorokiniana* was inoculated in a conical flask (250 mL) containing 97 mL PDB at an inoculation volume of 2%, then 1 mL of fermentation filtrate was added into and incubated with 180 r/min under 28 °C for 7 d. Sterile distilled water was used as a control. The fermentation broth was subsequently centrifuged in a 50 mL centrifuge tube at 5000 r/min for 15 min. The precipitated mycelia from each flask were collected and washed 3 times with sterile distilled water, then dried till the weight was constant at 60 °C and weighed. The spore germination rate was measured after incubation of *B. sorokiniana* in spore solution for 2 h.

### 4.5. Pot Control Tests

To evaluate the control efficacy of XZ34-1 on common root rot, pot experiments were carried out under greenhouse conditions. Qiule 2122, one of the most widely planted cultivars in Henan Province, China, was used. Before sowing, wheat seeds were surface disinfected with 0.5% NaClO and then washed with sterile distilled water. The seeds were sown on plastic pots (30 cm in diameter) filled with horticulture soil. Pot experiments were conducted in a greenhouse from 25 to 30 °C and relative humidity ranging from 30 to 70%. After 14 days, 10 mL of *B. sorokiniana* spore suspension (1 × 10^6^ cfu/mL) was poured around the roots of wheat in the pot. The roots of each pot of wheat were irrigated with 20 mL of CF after being inoculated with *B. sorokiniana*. Carbendazim (50% wettable powder) was used as a positive control, and water was used as the negative control. Each group of treatments had five replicates with at least 25 wheat plants in each replicate. Disease severity was evaluated on the 10th day after inoculation, and the scoring standard was 0 to 4. Level 0 indicates healthy plants, level 1 indicates incidence ≤25%, level 2 indicates incidence >25 to 50%, level 3 indicates incidence >50 to 75%, level 4 indicates incidence >75%. The disease incidence rate (DIR), disease index (DI), and control efficacy (CE) were using the following formulas [58].

DIR (%) = (n/N) × 100(1)

DI = ∑ [(Ni × i)/(N × 4)] × 100(2)

CE (%) = [(DIck − DIt)/DIck] × 100(3)

where n is the number of infected plants, N is the total number of investigated plants, Ni denotes the number of infected plants of a certain severity, i denotes a certain severity, 4 denotes the highest severity, DIck indicates the disease index of the control group, and DIt indicates the disease index of the treatment group.

### 4.6. Detection of Antifungal and Plant Growth-Promoting Traits

The plate test was used to detect antifungal and growth-promoting traits of strain XZ34-1. Indole acetic acid (IAA) production, Nitrogen-fixation production, phosphate solubilization, siderophore production, hydrolases, including cellulase, chitinase, protease and pectinase, were detected by the methods described by Ben Khedher et al. [32].

### 4.7. PCR Detection of Lipopeptide Biosynthesis Genes

Structural genes DNA of Bacyllomicin, Bacilysin, Bacillaene, Iturin, Fengycin and Surfactin in the antagonistic strain XZ34-1 was detected by PCR. Primers used to amplify the genes encoding for lipopeptides were summarized in Table 2. The PCR conditions were as follows: 94 °C for 5 min, followed by 32 cycles of 30 s at 94 °C, 30 s at 54–61 °C and 90 s at 72 °C, then a final elongation step of 10 min at 72 °C. The amplification products were detected by agarose gel electrophoresis (1.2% agarose, 120 V, 25 min), stained with ethidium bromide and then visualized under UV light. 

### 4.8. Effect of CF on Cell Membrane Integrity in B. sorokiniana

The effect of CF on cell membrane integrity was detected by propidium iodide (PI) staining [59]. *B. sorokiniana* (1 × 10^6^ cfu/mL) was inoculated into 40 mL of PDB and incubated at 28 °C for 72 h, and 10 mL of CF was added and incubated for another 12 h. LB liquid medium without CF was set as a control. Mycelia were collected by centrifugation at 8000 rpm/min for 10 min and washed twice with phosphate buffer (PBS), then resuspended in PBS and stained with PI (10 μg/mL) for 10 min in the dark. After staining, the mycelia with uniform thickness and even surfaces were washed twice and observed using a laser scanning confocal microscope (LSCM).

### 4.9. Detection of Antioxidant Activity of Wheat Root

Antioxidant Enzyme activities of SOD, peroxidase POD, PAL and MDA concentrations in the root tissue extracts of wheat subjected to different treatments were detected. Root extracts were prepared according to the method of Vaishnav et al. and Zhang W with minor modification [60]. Root tissues were ground in a mortar and pestle containing liquid nitrogen. The prepared powder was immersed in ice-cold 50 mM potassium phosphate buffer (pH 7.8) containing 1% polyvinylpyrrolidone (PVP) and 1 mM ethylene diamine tetra-acetate (EDTA). The homogenates were centrifuged at 8000 rpm at 4 °C for 20 min. The supernatants were immediately used to determine SOD, POD and PAL enzyme activities and MDA concentrations. In the case of every enzyme under detection, each treatment consisted of at least five replicates.

#### 4.9.1. SOD Activity

The activity of SOD was assessed by nitroblue tetrazolium (NBT) photoreduction according to the method of He S. Z. et al. with minor modifications [61]. The reaction mixture (3.0 mL) containing phosphate buffer (25 mM, pH 7.8), L-methionine (13 mM), riboflavin (2.0 × 10^-3^ mM), NBT (7.5 × 10^-2^ mM), EDTA (1.0 × 10^-2^ mM) and 0.1 mL of enzyme extracts were incubated at 30 °C for 25 min under cool-white fluorescent light at 4000 lx. The complete reaction medium incubated in the dark without the enzyme was used as the dark control. Samples were placed in the dark after the reaction had stopped, and then the optical density was measured at 560 nm.

#### 4.9.2. POD Activity

The activity of POD was measured by the modified method of Choudhary [62]. The reaction mixture was prepared by adding 6.9 mL of distilled water, 1.0 mL of 0.1% guaiacol and 1.0 mL of 0.18% hydrogen peroxide (the reference was replaced with 1.0 mL of distilled water) into 1.0 mL of the enzyme extract. The reaction was carried out at 30°C for 10 min then terminated through the addition of 0.2 mL of 5.0% metaphosphoric acid, and the absorbance was measured at 470 nm.

#### 4.9.3. PAL Activity

The activity of PAL was measured by the modified method of Shekhar Jain et al. [63]. Conversion of L-phenylalanine to cinnamic acid was performed at 30 °C. The reaction mixture was prepared by adding 2.0 mL of 100 mM Tris-HCl buffer (pH 8.8) and 1.0 mL of 20 mM phenylalanine into 1.0 mL of the enzyme extract. The reference used 1.0 mL of extraction buffer instead of the sample. The reaction was terminated through the addition of 0.2 mL of 6 M HCl 60 min after the start of the reaction, and the absorbance was measured at 290 nm. 

#### 4.9.4. MDA Concentration

The MDA concentration was determined by the method described by Anket Sharma et al. with slight modifications [50]. The reaction mixture consisted of 1.5 mL of 0.1% thiobarbituric acid (TBA) and 1.5 mL of root extract, which was reacted in a boiling water bath for 15 min and then rapidly cooled down and centrifuged at 9000 rpm for 5 min at 4 °C. The absorbance at 450 nm, 532 nm and 600 nm was determined using the supernatant.

### 4.10. Statistical Analysis

Each experiment was carried out at least three times. All data were expressed as mean ± SD and analyzed by one-way analysis of variance at the 5% level. Statistical differences between treatments were analyzed by Duncan’s multiple range test at a 5% significance level.

## 5. Conclusions

*B. amyloliquefaciens* XZ34-1 showed a high inhibition rate against pathogenic *B.* *sorokiniana* and had a broad antifungal spectrum. CF of XZ34-1 exhibited significant control effects on common root rot. Further analysis showed that *B. amyloliquefaciens* XZ34-1 had plant growth-promoting characteristics and could produce lipopeptides. Furthermore, activities of defense-related antioxidant enzymes in wheat seedlings were enhanced after inoculating with *B.* *sorokiniana* and treating with CF. Therefore, CF of XZ34-1 is a potential candidate for application as a biological control agent against *B.* *sorokiniana*.

## Figures and Tables

**Table 1:** Inhibition rates of mycelia growth of five aseptic filtrate against pathogens.

Strain	Inhibition Rate (%)				
	*Bipolaris* *sorokiniana*	*Fusarium* *graminearum*	*Rhizoctonia zeae*	*Aspergillus* *niger*	*Aspergillus* *flavus*
XZ34-1	53.52 ± 0.86 a	43.06 ± 1.73 a	46.70 ± 1.37 a	35.77 ± 1.22 a	34.35 ± 1.49 a
XZ33-1	32.66 ± 1.77 b	30.41 ± 0.75 b	33.93 ± 0.59 b	19.98 ± 2.84 b	26.94 ± 1.42 b
XZ22-1	22.02 ± 2.25 c	12.41 ± 1.77 d	26.70 ± 2.58 c	--	--
XZ19-2	15.86 ± 3.04 d	19.94 ± 1.17 c	26.25 ± 1.69 c	9.22 ± 1.62 c	8.73 ± 1.73 c
XZ11-2	15.17 ± 2.29 d	13.59 ± 2.42 d	12.64 ± 1.87 d	--	--

Values represent the means ± standard of at least nine replicates. Different letters indicate significant differences using Duncan’s multiple range test (*p* ≤ 0.05).

**Table 2:** Morphological and biochemical characteristics of XZ34-1.

Morphological and Biochemical Characteristics		XZ34-1
Cell morphology	Gram’s reaction	Gram positive
	Endospores	+
Colony morphology	Morphology	Rounded
	Pigment	Creamy white
	Surface	Rough
	Margin	Smooth
	Opacity	Opaque
Biochemical tests	Nitrate reduction	−
	Citrate utilization	−
	V-P test	+
	Methyl Red	−
	Glucose decomposition	Acids, not gas

“+” positive; “−” negative.

**Table 3:** Biocontrol efficacy of XZ34-1 CF on common root rot.

Treatments	Disease Incidence Rate (%)	Disease Index	Control Efficacy (%)
Sterile water control	98.05 ± 2.76 a	65.73 ± 11.29 a	−
Carbendazim treatment	41.24 ± 10.87 c	9.16 ± 2.20 b	85.67 ± 4.67
CF treatment	54.13 ± 9.16 b	14.17 ± 3.01 b	78.24 ± 4.66

The data presented are the mean values of five independent experiments. Standard errors of the mean values are presented after the ± symbol. Letters in the same column indicate DMRT at *p* < 0.05.

**Table 4:** Genetic markers used for detection of lipopeptide biosynthesis genes of XZ34-1.

Product	Genes	Primers	Primer Sequence (5′-3′)	Expected/Detected Size (bp)	References
Bacyllomicin	BMYBa	BMYBa-R	CGAAACGACGGTATGAAT	371/yes	Farzand, A. et al. [27]
		BMYBa-R	TCTGCCGTTCCTTATCTC		
	BMYBb	BMYBb-F	GAATCCCGTTGTTCTCCAAA	370/yes	Zhang, L. et al. [28]
		BMYBb-R	GCGGGTATTGAATGCTTGTT		
Bacilysin	BACa	BACa-F	ATCTTTATGGCGGCAGTC	595/yes	Farzand, A. et al. [27]
		BACa-R	ATACGGCTTACAGGCGAG		
	BACb	BACb-F	CAGCTCATGGGAATGCTTTT	498/yes	Mora, I. et al. [29]
		BACb-R	CTCGGTCCTGAAGGGACAAG		
Bacillaene	BAE	BAE-F	ATGTCAGCTCAGTTTCCGCA	688/yes	Compaore, C. S. et al. [30]
		BAE-R	GATCGCCGTCTTCAATTGCC		
Iturin	ITU	ITU-F	GGCTGCTGCAGATGCTTTAT	423/yes	Mora, I. et al. [29]
		ITU-R	TCGCAGATAATCGCAGTGAG		
Fengycin	fenD	FEND-F	TTTGGCAGCAGGAGAAGTTT	964/no	Athukorala, S. N. P. et al. [31]
		FEND-R	GCTGTCCGTTCTGCTTTTTC		
Surfactin	srfAA	srfAA-F	TCGGGACAGGAAGACATCAT	201/no	Khedher, S.B. et al. [32]
		srfAA-R	CCACTCAAACGGATAATCCTG		
	sfP	SFP-F	ATGAAGATTTACGGAATTTA	675/no	Chung, S. et al. [33]
		SFP-R	TTATAAAAGCTCTTCGTACG		

## Data Availability

Not applicable.
